# A Review on Antihyperglycemic and Antihepatoprotective Activity of Eco-Friendly *Punica granatum* Peel Waste

**DOI:** 10.1155/2013/656172

**Published:** 2013-06-25

**Authors:** Sushil Kumar Middha, Talambedu Usha, Veena Pande

**Affiliations:** ^1^Department of Biotechnology, Bhimtal Campus, Kumaun University, Nainital, Uttarakhand 263136, India; ^2^Department of Biotechnology & Biochemistry, Maharani Lakshmi Ammanni College for Women, Bangalore 560012, India

## Abstract

Over the past decade, pomegranate (*Punica granatum*) is entitled as a wonder fruit because of its voluminous pharmacological properties. In 1830, *P. granatum* fruit was first recognized in United States Pharmacopeia; the Philadelphia edition introduced the rind of the fruit, the New York edition the bark of the root and further 1890 edition the stem bark was introduced. There are significant efforts and progress made in establishing the pharmacological mechanisms of peel (pericarp or rind) and the individual constituents responsible for them. This review provides an insight on the phytochemical components that contribute too antihyperglycemic, hepatoprotective, antihyperlipidemic effect, and numerous other effects of wonderful, economic, and eco-friendly pomegranate peel extract (PP).

## 1. Introduction

The family Punicaceae contains a single genus, *Punica*, and two species the most predominant species is *Punica granatum *(pomegranate), and the less predominant is *Punica protopunica *(Socotran pomegranate), atypical to the island of Socotra.

In spite of its wide prehistoric background, the pomegranate has attained relatively few universally recognized names as mentioned in Table 1 [1].

### 1.1. Scientific Classification

Pomegranate (*Punica granatum*) has been placed under the subclass *Rosidae*, order *Myrtales*, along with other fruits such as guava and feijoa [2]. It is an evergreen or deciduous and spiny plant with multiple trunks and small slender leaves with tiny stems that is believed to have originated in Iran then moved to the Himalayas in northern India. Heterostylous funnel-shaped red flowers are characteristic to this plant and are found either in singles or in clusters of up to five [3]. The fruit is almost round in shape with a crown at the base created by the calyx. The skin is tough and leathery in texture, yellow or deep pink/red in color, and about 2 to 5 inches in width. The interior of the fruit contains white spongy membranous walls that form compartments containing sacs packed with a fleshy, juicy, red or whitish pulp. Each sac holds an angular, soft or hard seeds which are usually red or white in colour. About 52% of the mass, of the entire fruit is represented by these seeds [1].

Pomegranate peels or skin or rind (PP) are underestimated as an agricultural waste, though it is part of an ancient fruit with exceptionally rich ethnomedical applications and astringent properties. PP acts as ecofriendly waste because of its numerous uses such as reducing agent in making silver nanoparticles. PP also used for cattle feed and extraction of natural dyes [4]. 

### 1.2. Historical Uses of *Punica granatum*

**Figure 1:**
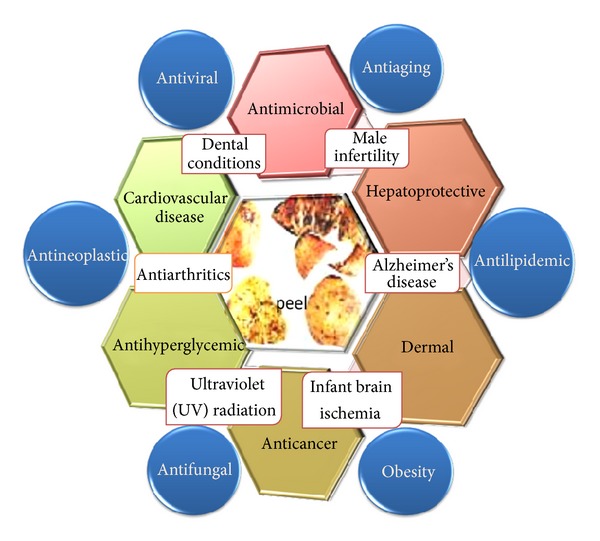
Principal therapeutic effects of pomegranate peel.

Many researchers have focused on the biological waste part of this wonder fruit, pomegranate, for the purpose of discovering many miraculous effects for human health. The potential therapeutic properties of PP are wide-ranging and include treatment and prevention for cancer [5, 6], cardiovascular disease [7], diabetes [8], dental conditions [9], and erectile dysfunction [10], protection from ultraviolet (UV) radiation [10], and antimicrobial [11]. Other potential applications include infant brain ischemia, Alzheimer's disease [8], male infertility, arthritis [10], dermal wounds [11], and obesity [10] (Figure 1).

In order to facilitate further investigation and utilization, we summarized the research achievements on phyto-chemical components that contribute for anti-hyperglycemic, antilipidemic, and hepato-protective effects of *Punica granatum* peel waste till date.

### 1.3. Proximate Physicochemical Composition/Nutritional Values of PP

Studies have shown that PP is highly nutritive and contains important raw materials like crude fibers, protein, and carbohydrates. The compositions of some of the ingredients are tabulated in Table 2 [12].

Another study showed that the chemical composition of pomegranate bagasse (the dried part of the fiber that remains once the juice is extracted) powder contains protein, fat, ash, total dietary fiber, insoluble dietary fiber, and soluble dietary fiber of 10.94, 20.86, 2.55, 50.29, 30.41, and 19.88 g/100 g, respectively [13]. However, the quantification of many components such as vitamins, minerals, and other pharmacological properties has to be evaluated in detail.

## 2. Phytochemicals/Active Constituents

**Figure 2:**
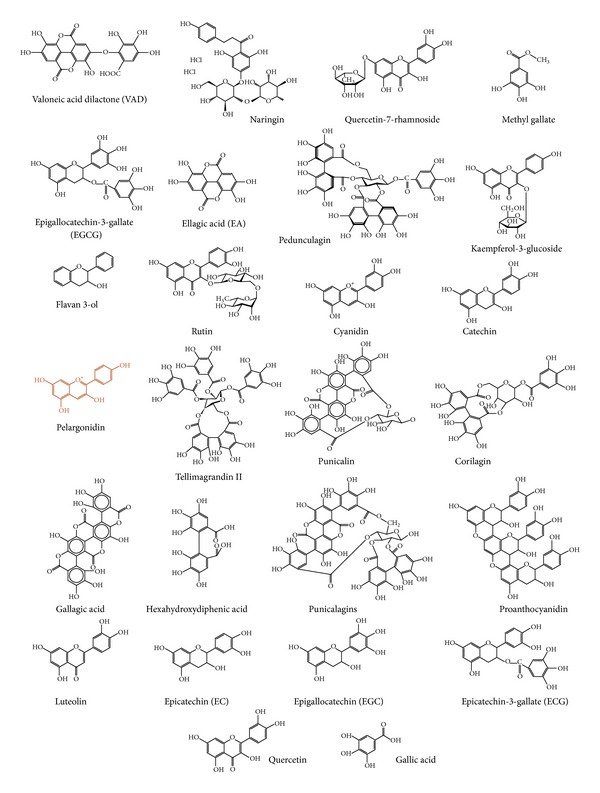
Structures of polyphenolic compounds found in pomegranate peel (*Punica granatum*) (figures were sketched using ChemSketch software alias Marvin Sketch).

During the ancient era, significant efforts and progress were made in establishing the pharmacological mechanisms of PP and the individual constituents responsible for them. PP is known to possess diverse phytochemicals, most of which are observed to have therapeutic properties. The major chemical constituents along with their bioactivity are tabulated in Table 3.  Figure 2 depicts the structure of all these chemical constituents of Table 3.

Punicalin and punicalagin are the major constituents of pericarp ranging up to 0.2% of the total amount. The brilliant red colour of peel is attributed to anthocyanidins and flavan-3-ols. Flavones and flavonols constitute the major flavonoids of peel. The methanolic extract of dried PP showed the presence of high content of phenolic compounds (44.0%) along with other constituents [34]. Phenolic acids such as caffeic acid, fumaric acid, chlorogenic acid and p-coumaric acid are present in the pericarp [17]. The amount of ellagic acid in fruit peel of 12 varieties examined by Akbarpour et al. [33] fluctuates considerably with a maximum of 50 mg/100 g (Syah-e-saveh) and a minimum of 10 mg/100 g (Rabbab and Shishe-Kap) (Table 4). Gil et al. [23] reported that the amount of total phenolics in peel was evidently higher than arils of pomegranate fruit [33, 35].

Ben Nasr et al. [36] have described that PP has ellagic acid (EA), ellagitannins and gallic acid. PP contains hydroxybenzoic acids such as gallagic acid, EA, and EA glycosides [17]. Anthocyanidins are principally cyanidin, pelargonidin and delphinidin [26] and flavonoids such as kaempferol, luteolin, and quercetin [27]. Murthy et al. [37] quantified methanolic extract of PP using HPLC and reported the presence of gallic acid (34.03%) and catechin (3.31%).

Studies on PP were undertaken to investigate the changes in the major chemical composition during fruit maturation in two Israeli commercial varieties, “Wonderful” and “Rosh-Hapered”. The result of the study revealed the levels of total phenolic antioxidant activity and hydrolysable tannins were reduced during maturation, while the anthocyanin level increased. This knowledge could help establish the optimum harvest date ensuring the maximum nutritional properties of PP [38]. The results of Turkish researchers showed that the levels of total phenolic compounds changed depending on cultivars and fruit parts. In all cultivars, the highest levels of total phenolic content were obtained from the peel extracts.

## 3. Physical Properties

Twelve pomegranate (*Punica granatum* L.) cultivars from different regions of Iran were analyzed for their physical properties by Akbarpour et al. [33]. The amount of EA ranged 10–50 mg/100 g in different varieties of PP. The highest amount of EA was found in Syah-e-Saveh variety (50 mg/100 g). 

## 4. Validated Pharmacological Properties of Pomegranate Peel

### 4.1. Antioxidant Activity

Superfluous generation of the free radicals is proved to instigate and aggravate many human ailments like arthritis, cancer, Alzheimer's disease, Parkinson's disease, AIDS and diabetic complications [51, 52]. Reports indicate that plants rich in anthocyanin, flavonoids and polyphenols are observed to be effective in scavenging the free radicals [53, 54]. Total antioxidant activity of 12 different varieties was determined by FRAP (ferric reducing antioxidant power) method described by Akbarpour et al. are enlisted in Table 4 and it ranged from 225.17 to 705.50 mmol/g. According to Akbarpour et al. [33] study, the highest antioxidant activity level was detected in “Lamsari-e-Behshahr” and the lowest in “Naderi”. The same study has revealed that the antioxidant activity of peel is higher than that of juice and this difference is attributed to the presence of pomegranate peel tannins [38].

The first report on antioxidant property of PP using *in vitro* models was elucidated by Singh et al. [55]. The methanolic extract of peels showed 83% and 81% antioxidant activity at 50 ppm using the *β*-carotene-linoleate and 2,2-diphenyl-2-picrylhydrazyl (DPPH) model systems, respectively. Similar group also showed 56, 58, and 93.7% inhibition using the thiobarbituric acid (TBA) method, hydroxyl radical scavenging activity, and serum low-density lipoprotein LDL oxidation, respectively, at 100 ppm [56]. Owing to this property, the studies can be further extended to exploit PP for their possible application in the preservation of food products as well as their use as health supplements and nutraceuticals.

Additional studies have also shown that PP had the highest antioxidant activity among the peel, pulp, and seed fractions of 28 kinds of fruits commonly consumed in China as determined by FRAP assay [57]. A mixture of ethanolic, methanolic and acetone PP extract had markedly higher antioxidant capacity than the pulp extract in scavenging capacity against superoxide anion, hydroxyl, and peroxyl radicals as well as inhibiting CuSO_4_-induced LDL oxidation. The contents of total phenolics, flavonoids and Proanthocyanidins were also higher in peel extract than in pulp extract. The large amount of phenolics contained in peel extract may contribute to its strong antioxidant ability [46].

Al-Mustafa and Al-Thunibat [58] have revealed the antioxidant activity of some Jordanian medicinal plants used traditionally for treatment of diabetes. They were categorized PP under high antioxidant potential plants. The activity investigated in methanolic and aqueous extract was DPPH-TEAC IC_50_ 365 ± 5.0 mg/g GAE, 2,2′-azinobis-(3-ethylbenzothiazoline-6-sulfonic acid) (ABTS)-IC_50_ 6.9 ± 0.1 *μ*g/mL; and DPPH-TEAC IC_50_ 267.1 ± 3.5 mg/g GAE; and ABTS^+^-IC_50_ 9.8 ± 0.1 *μ*g/mL, respectively. Among all the plants chosen by traditional healers, methanolic and aqueous extracts of PP both were having potent radical scavenging activity. Methanolic extract of PP exhibited the highest phenolic content (103 GAE mg/g) among all their experimental plants.

In a recent study [59], the antioxidant activity of methanolic extract of PP on brain of normal rats demonstrated that it reduced lipid peroxidation and nitric oxide in both serum and brain tissue homogenate, having an effect on the scavenging capacity of superoxide anion and hydrogen peroxide.

Pan et al. [60] described a continuous (CUAE) and pulsed ultrasound-assisted (PUAE) method for the extractions of antioxidants from PP since there is a great demand for developing efficient extraction methods in order to reduce extraction time and increase the yield and activity of functional antioxidants. Cumulatively, all these observations suggest the potential antioxidant activity of PP (Table 5).

### 4.2. Antihyperglycemic Effect

Over the past decade, significant progress has been made in establishing the anti-hyperglycemic pharmacological mechanism of PP and the individual compounds responsible for it. Various solvent extracts of PP appear to have anti-diabetic property. On their Jordanian medicinal plants survey, native authors have revealed 61% traditional healers recommend PP for diabetes treatment [58]. Similar kind of studies done by the authors in India also revealed the uses of PP by traditional healers (vaidya) (49%) and anti-hyperglycemic effect using *in vitro* glucose oxidase method [61].

The first report on the antidiabetic properties of PP using an *in vivo* model was elucidated by Nogueira and Pereira, followed by Zafar and Singh and Nozire and Serpil, reproduced by several other studies [47–50, 62–66].

Diabetic rats treated with 0.43 g/kg B.W. of aqueous peel extract for four weeks displayed significantly lowered blood sugar level and increased number of *β* cells which relatively help in intensification of insulin level [47]. The mechanistic anti-diabetic activity of the extract is by stimulation, regeneration, and increased number of *β* cells, by protecting pancreatic tissue and subsequent release of insulin. Also, it may increase the stimulation and activation of insulin receptor [47].

Oral administration of aqueous extract of PP at doses of 50 and 100 mg/kg for 21 days brought down the fasting blood glucose, total serum cholesterol (TC), triglycerides (TG), serum low density lipoprotein cholesterol (LDL-c), VLDL-C (very low-density lipoprotein cholesterol) and tissue LPO (lipid peroxidation) levels together with boost on high-density lipoprotein cholesterol (HDL-c), GSH (glutathione) content and antioxidant enzymes in contrast with diabetic control group. These authors recommended the uses of aqueous extract of the peel as a dietary supplement in the cure and inhibition of chronic diseases categorized by aggravated antioxidant status and diminished glucose metabolism [48]. 

The same group emphasized on oral administration of the aqueous-ethanolic extract of PP which led to significant blood glucose lowering effect in normal, glucose-fed hyperglycemic in their another report on alloxan-induced diabetic rats. This report suggested that the effect can be due to increased peripheral glucose utilization or retardation of intestinal glucose absorption may also be partly responsible for inhibition of hyperglycaemia in glucose-fed rats [49].

Althunibat et al. [50] had shown in their study on STZ-(streptozotocin-) induced diabetic rats that intraperitoneal (*i.p*.) administration of 10 and 20 mg/kg BW of PP for four weeks significantly enhanced the activities of antioxidant enzymes in liver, kidney, and RBC. The methanolic extract of PP (75 and 150 mg/kg, daily) inhibits glucose level in alloxan-induced diabetic *wistar *rats. The extract also caused a significant reduction in malondialdehyde (MDA), a lipid peroxides marker, in diabetic rat tissues and elevated the total serum antioxidant capacity in a dose-dependent manner. Phytochemical investigation demonstrates that the gallic acid in the methanolic extract of peels is mainly responsible for this activity [8]. This group suggested that the effect may be due to the presence of terpenoids such as ursolic acid and oleanolic acid, which may help to scavenge the free radicals generated during diabetes (Table 6). 

### 4.3. Antihyperlipidemic Effect

Alterations in lipid profile are one of the most common complications in diabetes mellitus and affects 40% of all diabetic patients [51]. A comparative study was carried out by Cheng et al. (2004) [67] to elucidate the hypolipidemic effect of crude PP and polyphenolic extract. Male Sprague-Dawley (SD) rats were fed with a high-fat diet to induce hyperlipidemia and the treatment was carried out for 28 days. The results revealed decrease in the levels of TC/HDL-c ratio and serum LDL-c levels concluding PP polyphenolic extract is effective in lowering serum and hepatic lipids (Table 6).

### 4.4. Hepatoprotective Effects

Recent *in vivo* animal studies have evaluated the hepatoprotective effects of pomegranate, however, the exact mechanism and significant compounds have not yet been described. 

The effects of continuous administration of PP on experimentally induced liver fibrosis in rats have been examined by Toklu et al. [68]. The levels of serum aspartate aminotransferase (AST), lactate dehydrogenase (LDH), and alanine aminotransferase (ALT) were ascertained in order to evaluate liver functions and the amount of tissue damage. The AST, LDH, ALT, and cytokine levels in the serum, which were elevated in liver fibrosis models, were found to be considerably reduced and brought to near-normal levels after PP treatment. Similarly, the increase in hepatic collagen levels was also diminished leading to an improvement in the structure and functions of the liver. It was thus ascertained that PP possesses certain hepato-protective properties making it an important therapeutic agent in the treatment of fibrosis and oxidative damage [68, 69]. 

Previous work showed that feeding of rats with PP provided protective effect against carbon tetra chloride (CCl_4_) toxicity [55]. Studies in rats with CCl_4_-induced liver damage demonstrated pretreatment with PP enhanced or maintained the free radical scavenging activity of the hepatic enzymes catalase, superoxide dismutase, and peroxidase and resulted in 54% reduction of lipid peroxidation values compared to controls confirming the antioxidant property of the PP [50].

A discrete investigation in rats with CCl_4_-induced liver damage demonstrated pretreatment with PP extract boosted the free radical scavenging activity of the hepatic enzymes superoxide dismutase, and catalase, and showed 54% decline in lipid peroxidation as compared to controls [69].

Hepatocellular carcinoma (HCC), a common and fatal cancer, is majorly driven by oxidative stress. The effect of pomegranate emulsion (PE) on DENA-induced hepatocarcinogenesis, which mimics HCC in humans, was examined in an animal model. Robust chemopreventive activities were reported owing to reduction in the incidence, size, volume and multiplicity of the hepatic nodules. In addition, PE also alleviated lipid peroxidation and oxidation of proteins in the liver. The report thus suggests and supports the use of pomegranate-derived agents in the treatment and prevention of HCC in humans [70].

## 5. Mechanisms of Action

A recent study by Jain et al. [32] has shown the probable mechanism of action of PP. They have isolated and purified a compound Valoneic acid dilactone (VAD) from the methanolic extract of *Punica granatum *(MEPG). MEPG-(400, 370 mg/kg, p.o.) and VAD-(25, 50 mg/kg, p.o.) treated groups have shown very minimal acinar damage and adequate number of pancreatic islets. Their findings suggest that some pancreatic *β*-cells are still surviving to exert their insulin releasing effect. In diabetic condition, weight loss arises due to the impairment in insulin action in the conversion of glucose into glycogen. The catabolism of fats does not occur as there is an inhibition of lipolysis due to its unavailability which is because of destruction in beta cells. VAD and MEPG were found to inhibit the alpha amylase activity which is a carbohydrate-hydrolysing enzyme, thus decreases postprandial hyperglycemia and improves glucose metabolism. The extracts VAD and MEPG were found to inhibit PTP1B activity which showed increase in insulin receptor tyrosine phosphorylation which mimicked *in vivo* action of insulin thereby decreased plasma glucose levels in a dose-dependent manner. The findings of their studies such as, blood glucose levels, oral glucose tolerance test, body weight, mortality and histopathology were in correlation with each other and indicate that MEPG and VAD could be beneficial in management of diabetes and associated complications. VAD was observed to be a more powerful inhibitor of aldose reductase, *α*-amylase, and protein tyrosine phosphatase PTP1B, when compared to MEPG because of its polyphenolic nature.

## 6. Toxicity Studies/Safety/Adverse Effects

There are relatively none *in vivo* or *in vitro* studies reporting the toxic or adverse effect of the PP on any of the mammalian system. The LD_50_ of the aqueous extract of PP in mice was found to be 1321 ± 15 mg/kg *i.p.* [71].

## 7. Conclusion

Industries have moved towards usage of naturally derived compounds, biologics as they are easily extractable and can be cultured in labs. Amongst these sources, ecofriendly [4] *Punica granatum *peel has showed effectiveness due to the array of compounds present in it. These compounds have good antioxidant activity and also serve as excellent nutritional supplements. This review highlights the work that has been done in the recent past on the PP, which includes anti-hyperglycemic [8], hepatoprotective [56], and anti-hyperlipidemic effects [67]. These experiments have shown productive results both in *in vivo* and *in vitro* domains. Hence more labs are moving towards PP as a suitable therapeutic agent in various diseases. 

## 8. Future Direction

Despite the documented roles in nutrition, there are very few phytochemical studies. The actions of mechanism of these compounds are still not very clearly understood, however, it is predicted that the mechanisms are quite complicated since there may be numerous factors involved in it.

There is a dearth in the field of pharmacodynamics and pharmacokinetics or safety aspects of the PP. There have been very few studies pertaining to the molecular aspects of PP, which is clearly evident by the sequence of information available in the public domains. Even though PP is rich in healing properties, due to the lacunae in many aspects, there is an urgent requirement for further investigations to delineate its precise mechanisms and possible therapeutic values, particularly in the field of diabetes mellitus.

## Figures and Tables

**Table 1:** Few commonly renowned vernacular names of *Punica granatum*.

Country	Recognized name
Roman	Carthage (*Punica*)
Italian	Melogranato, melogranogranato, pomogranato, or pomopunico
Spanish	Granada (the fruit), granado (the plant)
Dutch	Granaatappel
French	Grenade
German	Granatapfel
India	Dadima or dalima or dalim or Anar
Persian	Dulim or dulima
Guatemala	Granad
Indonesia	Gangsalan
Samoan	Limoni
Brazilian	Roma, romeira, or romazeira
Thailand	Tab tim
Malaya	Delima

**Table 2:** Nutrient content of pomegranate peel per 100 g [12].

Composition	Content
Total solid	94.50
Moisture	5.40
Total sugars	17.70
Reducing sugars	4.34
Protein	4.90
Crude fiber	16.30
Fat content	1.26
Ash	3.40

**Table 3:** Active constituents and their biological activity of *Punica* peel.

Compound	Bioactivity	Reference
Major tannins of pomegranate peel		

Casuarinin	Antiviral, antioxidant	[14]
Corilagin	Antihypertensive, antineoplastic	[14–16]
Ellagic acid [EA]	Antineoplastic, skin whitening	[17, 18]
Gallic acid	Antimutagenic, anti-inflammatory, antiviral, antioxidant	[17, 19]
Methyl gallate	Antioxidant	[20]
Granatin A	Antioxidant, anti-inflammatory	[21]
Granatin B	Antioxidant, anti-inflammatory	[21, 22]
Pedunculagin	Antineoplastic, antioxidant	[14]
Punicalagin	Antioxidant, antihypertensive, anti-hyperglycemic	[22–24]
Punicalin	Antioxidant, anti HIV, anti-hyperglycemic	[21–23]

Major flavonoids of pomegranate peel		

Catechin	Antineoplastic, antioxidant	[25]
Cyanidin	Antioxidant	[26]
Epicatechin	Antineoplastic	[25]
Epigallocatechin 3-gallate	Antineoplastic	[25]
Flavan-3-ol	Antineoplastic	[25]
Kaempferol	Antioxidant, anti-inflammatory	[27]
Kaempferol-3-0-glucoside	Antioxidant	[27]
Kaempferol-3-0-rhamnoglycoside	Antihypertensive	[27]
Luteolin	Antioxidant, antioxidant	[27]
Luteolin-7-0-glucoside	Antioxidant	[27]
Naringin	Antiviral, antibacterial	[28]
Pelargonidin	Antiviral, antibacterial	[26]
Quercetin	Antiviral, antioxidant, antineoplastic	[29]
Rutin	Antiviral, antioxidant, antihypertensive	[29]

Major alkaloids of pomegranate peel		

Pelletierine	Antioxidant	[30, 31]
Valoneic acid dilactone	Antidiabetic	[32]

**Table 4:** Amount of EA in different varieties of peel [33].

S. no	Variety	Thickness (mm)	Antioxidant activity (mmol/100 g)	Amount of ellagic acid (mg/100 g)
1	Rabbab	6.01	229.67	10.00
2	Malas-e-Yazd	4.06	234.33	30.00
3	Malas-e-Saveh	1.92	440.50	20.00
4	Shishe-Kap	2.53	303.83	10.00
5	Khazar-e-Bardeskan	2.10	640.17	25.00
6	Naderi	2.91	225.17	30.00
7	Alak	2.62	463.50	20.00
8	Abdandan	2.13	284.93	37.75
9	TabriziPoost	2.57	374.38	32.50
10	Syah-e-Saveh	1.60	314.89	50.00
11	Syah-e-Badrood	2.33	327.63	40.00
12	Lamsari-e-Behshahr	1.727	705.50	45.00

**Table 5:** Overview of antioxidant pomegranate studies.

Assays	Effect	Reference
Trolox Equivalent Antioxidant Concentration (TEAC)	Aqueous extract Prodelphinidinshowed active antioxidant activity	[39]
Phospho-molybdenum complex	Methanolic extract revealed lowest antioxidant activity followed by ethyl acetate, acetone, and water extract	[40]
DPPH and ABTS radicals	Remarkable free radical-scavenging power	[41]
DPPH and ABTS radicals	Robust antioxidant activity	[42]
DPPH and ABTS radicals	Extraordinary free radical-scavenging power	[43]
FRAP	Peel had superior antioxidant activity than pulp and seed	[44]
FRAP	Seed exhibited lower antioxidant activity as compared to peels	[45]
DPPH and ABTS radicals	Methanolic extract of PP exhibited the highest phenolic content and free radical-scavenging power	[46]

**Table 6:** Overview of *in vivo Punica* peel studies for diabetes and its complications.

Preclinical trial	*in vivo* studies	Day/week	Nature of peel extract/dosages (per kg BW/day)	Outcome	Statistically significant results?	Reference
Diabetes	Albino Wistar rats	28/4	Aqueous/0.42 g	Lowering of blood sugar level and increased number of *β* cells	Yes	[47]

Diabetes	Albino Wistar rats	10/1.3	Methanolic/200 mg	Increase of the activities of antioxidant enzymes catalase, superoxide dismutase, glutathione peroxidase, glutathione-S-transferase, and glutathione reductase, in liver and kidney	Yes	[48]

Diabetes	Albino Wistar rats	15/2.3	Methanolic/200 mg	Improvement of hepatic, cardiac, and renal LPO and serum T3, T4, insulin, and glucose concentrations	Yes	[49]

Hyperlipidemic	Sprague-Dawley rats	28/7	Methanolic/5%, 10%, 15%	Reduction of total cholesterol, LDL-cholesterol, triglycerides. VLDL-cholesterol H	Yes	[50]

Diabetes	Albino Wistar rats	28/7	Methanolic/10 mg, 20 mg	Increase of the activities of antioxidant enzymes catalase, superoxide dismutase, glutathione peroxidase, glutathione-S-transferase, and glutathione reductase, in liver and kidney	Yes	[50]

Diabetes	Albino Wistar rats	42/6	Methanolic/75 mg and 150 mg	Increase of the activities of antioxidant enzymes catalase, superoxide dismutase, glutathione peroxidase, and decreased LPO level in hippocampal and cortex region of brain	Yes	[8]

Diabetes	Albino Wistar rats	21/3	Methanolic/200 mg and 400 mg	Reduction of aldose reductase, amylase, PTP1B inhibition assay	Yes	[32]
